# Global and item-level MADRS differences between melancholic and unspecified depressive symptoms under rTMS: an exploratory analysis

**DOI:** 10.3389/fpsyt.2026.1794892

**Published:** 2026-07-24

**Authors:** Carolina Viegas, Setareh Ranjbar, Jean-Frédéric Mall, Pierre Vandel, Armin von Gunten, Beatriz Pozuelo Moyano, Kevin Swierkosz-Lenart

**Affiliations:** 1 Service of Old Age Psychiatry, Department of Psychiatry, Lausanne University Hospital and University of Lausanne, Prilly, Switzerland; 2 Center for Research in Psychiatric Epidemiology and Psychopathology, Department of Psychiatry, Lausanne University Hospital and University of Lausanne, Prilly, Switzerland

**Keywords:** depression, MADRS, melancholic, rTMS, unspecified

## Abstract

**Background:**

Repetitive transcranial magnetic stimulation (rTMS) has demonstrated efficacy in treating major depressive disorder (MDD), but evidence regarding its use across the different depressive subtypes remains limited.

**Objective:**

To observe changes in overall MADRS scores and individual MADRS items in patients diagnosed with melancholic depressive features versus those with non-melancholic and non-atypical depressive features following rTMS.

**Methods:**

An exploratory prospective study was conducted involving 21 participants presenting with depressive symptomatology either with melancholic (n=11) or unspecified features, meaning non-melancholic and non-atypical (n=10). All subjects underwent a standardized 15-session rTMS protocol. Depressive symptoms were assessed using the Montgomery–Åsberg Depression Rating Scale (MADRS) before and after treatment.

**Results:**

After adjusting for confounding factors, an association was observed between the unspecified group and the greater reduction in the overall MADRS score (B = –9.08, 95% CI –17.69 to –0.46, p = 0.040). Significant results were also found for individual items of inner tension (B = –2.05, 95% CI –3.57 to –0.52, p = 0.012) and inability to feel (B = –1.94, 95% CI –3.10 to –0.78, p = 0.003).

**Conclusions:**

Our exploratory analyses show that patients with unspecified depressive features experience greater rTMS-related symptom improvement compared to those presenting melancholic features. These observations underscore the potential relevance of depressive subtypes in treatment response and warrant confirmation in larger, controlled studies.

## Introduction

Major depressive disorder (MDD) has a lifetime prevalence of approximately 15–18% and is a heterogeneous condition in terms of symptomatology, clinical course and response to pharmacological treatment (1). The disorder is frequently recurrent and a substantial proportion of patients fail to achieve adequate response to standard treatments (2). Data from large clinical cohorts, namely the STAR*D study, indicate that approximately 30% of patients do not achieve remission despite four treatment steps (3). Moreover, the risk of recurrence may reach up to 80% (4, 5).

Subtypes of depression are described in DSM-5, such as the melancholic subtype, the atypical subtype, the psychotic subtype and the anxious subtype, each one possibly associated with distinct biological mechanisms (6, 7).

The chronic, recurrent and potentially treatment-resistant nature of MDD highlights the need for novel therapeutic strategies, particularly neuromodulation techniques.

Repetitive transcranial magnetic stimulation (rTMS) modulates cortical excitability through the application of a magnetic field generated by a coil placed over the scalp (8), with low-frequency stimulation (≤1 Hz) transiently inhibiting regional activity, while high-frequency stimulation (≥5 Hz) typically exerts excitatory effects (9). In the treatment of depression, rTMS is most commonly applied as high-frequency excitatory stimulation targeting the left dorsolateral prefrontal cortex (DLPFC) (10, 11).

rTMS is a non-invasive neuromodulation technique and is considered safe. Nevertheless, it carries potential side effects. The most serious, albeit rare is the induction of seizures; scalp discomfort, tension-type headaches, tingling sensations and temporary auditory sensitivity due to the clicking sounds produced by the device are the most commonly reported (12).

Accurate and sensitive outcome measures are essential for evaluating the clinical efficacy of rTMS. The Montgomery–Åsberg Depression Rating Scale (MADRS), a widely used clinician-rated scale, enables assessment of overall depressive symptom severity (13) and provides detailed information at the individual item level, facilitating the characterization of depressive symptom profiles, thereby enabling more personalized and targeted interventions.

The aim of this study is to explore changes in the global MADRS score and its individual items in patients who have undergone rTMS treatment and to compare those diagnosed with melancholic depressive symptoms versus those with non-melancholic and non-atypical depressive symptoms. For the purposes of this article, this latter group will be designated as presenting unspecified depressive symptoms. However, this definition does not correspond to the unspecified depressive disorder subtype described in the DSM-5. Rather than grouping all non-melancholic presentations together, this approach was adopted to enhance clinical and phenotypic specificity. Other depressive subtypes, including atypical and psychotic presentations, were not examined due to insufficient sample sizes for meaningful analysis. This approach may reveal subtle clinical changes that could otherwise be overlooked when assessing only total score reductions and combining all depressive subtypes.

In fact, melancholic symptoms in MDD are associated with abnormal functional connectivity between the left DLPFC and the default mode network, particularly reduced anticorrelation between the left DLPFC and the precuneus/posterior cingulate cortex (14, 15). This connectivity dysfunction results in impaired top-down regulation and a dominant state of the posterior cingulate that perpetuates rumination and core depressive symptoms (15). Recent neurofeedback evidence demonstrates that normalizing left DLPFC–precuneus connectivity reduces depressive symptoms and rumination in patients with MDD (14) and this connectivity pattern has also been identified as abnormal in melancholic depression (15), highlighting its relevance as a network-level dysfunction in this subtype.

High-frequency rTMS applied to the left DLPFC represents a theoretically grounded intervention for this depressive subtype, as it increases cortical excitability (9) and has been shown to modulate dysfunction within and between large-scale functional networks, including the default mode and central executive networks, with such normalization associated with symptom improvement (16). Because this circuit-level rationale specifically implicates the DLPFC–precuneus abnormality that characterizes melancholic depression, it would predict that high-frequency left DLPFC rTMS engages mechanisms of particular relevance to this subtype. On this basis, and rather than assuming a uniform antidepressant effect across presentations, we set out to examine whether rTMS exerts a selective, subtype-dependent effect, by comparing treatment response between patients with melancholic and non-melancholic (unspecified) depressive features.

To our knowledge, no previous study has specifically addressed differential symptomatic changes between depression subtypes following rTMS. The present work aims to provide preliminary observations that may help generate hypotheses for future research.

## Methods

This prospective exploratory study included a sample of 21 individuals who received rTMS as part of routine clinical care for clinically significant depressive symptoms. All participants underwent a standardized clinical assessment and provided informed consent for the prospective collection and research use of clinical data.

We reviewed medical records of patients who presented to the Interventional Unit of the Old Age Psychiatry Service at Lausanne University Hospital (CHUV) with depressive symptoms and received rTMS treatment between January 2023 and December 2024.

Patients had to be 18 years of age or older. Inclusion was based on the presence of clinically significant depressive symptomatology, regardless of the underlying psychiatric diagnosis, reflecting a symptom-focused rather than a diagnosis-driven approach to evaluate change in MADRS scores under rTMS. No predefined MADRS cut-off was applied, as the focus was placed on clinical evaluation. In line with this approach, the indication for rTMS was based on a comprehensive psychiatric assessment rather than on symptom severity scores alone. All patients were receiving ongoing pharmacological treatment at the time of rTMS prescribed by their psychiatrist. Continuation of pharmacotherapy was encouraged and adjusted as needed according to the patients’ clinical presentation throughout the study. This naturalistic design reflects real-world clinical conditions, particularly in chronic or treatment-resistant presentations, where residual depressive symptoms, despite modest rating-scale scores, may be associated with substantial functional impairment and reduced quality of life (17, 18).

Informed consent was obtained either through CHUV’s general consent procedure (n = 14) or via oral consent during follow-up contact by the study team (n = 7). Exclusion criteria comprised failure to provide informed consent or incomplete data for key study variables. The study was approved by the Medical Ethics Committee of the Canton of Vaud (CER-VD).

Demographic and clinical data were collected, including age, sex, height, weight and the use of antidepressant and mood stabilizer medication. Melancholic and atypical features were systematically assessed in all patients using DSM-5 criteria. Melancholic features are defined by the presence of either a marked loss of interest or pleasure in nearly all activities or a lack of reactivity to usually pleasurable stimuli, in addition to at least three of the following: a qualitatively distinct depressed mood characterized by profound despondency, hopelessness or pervasive emptiness; diurnal mood variation with worsening in the morning; early morning awakening (≥2 hours earlier than usual); marked psychomotor agitation or retardation; significant appetite loss or weight reduction and excessive or inappropriate guilt (19). Atypical features are defined, according to DSM-5 criteria, by the presence of mood reactivity (i.e., mood brightening in response to actual or potential positive events) together with at least two of the following: significant weight gain or increase in appetite; hypersomnia; leaden paralysis (a heavy, leaden sensation in the arms or legs) and a long-standing pattern of interpersonal rejection sensitivity resulting in significant social or occupational impairment (19). In our cohort, the presence of psychotic features represents a criterion for non-referral to rTMS; consequently, no patient with psychotic depression was included in the treated sample. Moreover, no patient met DSM-5 criteria for atypical features. The unspecified group, therefore, comprised exclusively non-melancholic patients without atypical presentations. Because the anxious distress specifier can accompany either presentation, it was not used to define the study groups; patients were classified solely according to the presence or absence of melancholic features. Participants with anxious features were assigned to groups according to their melancholic status, resulting in 8 individuals with anxious features in the melancholic group and 7 in the unspecified group. Classification was based on a structured clinical assessment, including specific questions to address each DSM-5 criterion. Patients were subsequently classified as having melancholic depressive symptoms or unspecified depressive symptoms.

Among patients identified as presenting depressive symptoms and who underwent the 15-session rTMS protocol targeting the left DLPFC, three were excluded from the analysis, one due to the absence of a baseline MADRS score, one because depressive symptom severity was assessed using the Hamilton Depression Rating Scale instead of the MADRS and one due to the lack of informed consent.

Depressive symptom severity was assessed using the MADRS at baseline and following completion of the rTMS treatment protocol. The MADRS was administered as a clinician-rated interview by experienced clinicians or a trained research scientist as part of routine clinical practice, with items rated based on the patient’s symptoms over the preceding week. Post-treatment scores were obtained either during routine follow-up assessments or, when a contemporaneous MADRS was unavailable, reconstructed from detailed clinical documentation by an experienced rater applying the MADRS item anchors, which is acknowledged as a limitation.

rTMS was administered over the left DLPFC, with precise coil positioning guided by the Brainsight neuronavigation system. Stimulation was delivered at 20 Hz frequency (20), with 1,200 pulses per session (21). The stimulation intensity was set at 80% of the individual resting motor threshold (RMT) for most participants. In two cases, lower intensities of 55% and 60% of maximal stimulator output were used, as the patients did not tolerate higher intensities (22). Treatment consisted of 15 sessions administered over a 3-week period (23).

### Statistical analysis

Baseline characteristics of the sample were evaluated using descriptive statistics: mean (SD) for continuous variables and count (percentage) for categorical variables. Baseline characteristics were subsequently compared between participants with melancholic and unspecified depressive symptoms using the Wilcoxon test for continuous data and Fisher’s exact test for categorical data.

Changes in MADRS total scores and its 10 individual items were calculated for each participant between baseline and post treatment assessment following 15 rTMS sessions over a 3-week period follow-up. To explore whether these changes differed by depressive subtypes, namely melancholic or unspecified, we first performed a crude comparison using the Wilcoxon test. We then constructed separate multiple linear regression models for each outcome to obtain adjusted estimates. All models were controlled for potential confounders, including sex, age, administration of antidepressant and mood stabilizer medication and the baseline value of the respective dependent variable. For the regression analyses, results are reported as both unstandardized (original) β coefficients and standardized β coefficients to reflect effect size, each accompanied by their respective 95% confidence intervals.

All statistical analyses were conducted using the R software environment for statistical computing (Version 4.1.0). Statistical significance was predetermined at p ≤ 0.05 and no corrections for multiple comparisons were applied given the exploratory nature of the analyses.

## Results

The study sample consisted of 21 participants, with a mean age of 64.10 ± 15.95 years old. Eleven individuals met criteria for melancholic depressive symptoms, while ten were classified as having unspecified depressive symptoms. Females accounted for 57% of the cohort. However, the differences in the baseline characteristics were not statistically significant between the two groups (**Table 1**).

**Table 1:** Descriptive statistics.

Baseline characteristics	Overall N = 21*^1^*	Depression Subtypes		p-value*^2^*
		Melancholic N = 11*^1^*	Unspecified N = 10*^1^*	
Age	64.10 (15.95), 32.00-86.00	68.82 (10.64), 49.00-86.00	58.90 (19.56), 32.00-81.00	0.34
Sex				0.67
Female	12 (57%)	7 (64%)	5 (50%)	
Male	9 (43%)	4 (36%)	5 (50%)	
Height	1.67 (0.09), 1.48-1.80	1.70 (0.07), 1.58-1.80	1.62 (0.10), 1.48-1.73	0.16
Weight	70.44 (19.49), 43.00-120.00	72.80 (21.18), 43.00-120.00	66.50 (17.38), 45.00-92.00	0.66
Antidepressant	17 (81%)	9 (82%)	8 (80%)	>0.99
Mood stabilizer	5 (24%)	2 (18%)	3 (30%)	0.64
MADRS	27.43 (9.25)	27.73 (6.20)	27.10 (12.13)	0.67

1 Mean (SD); n (%).

2 Wilcoxon rank sum test; Fisher’s exact test.

Regarding pharmacological treatment, 17 participants were receiving at least one antidepressant at the start of the rTMS protocol and five participants were on mood stabilizers (**Table 1**).

Overall, MADRS scores at baseline ranged from 20 to 48 and 3 patients had baseline scores below this threshold (12, 13 and 18). Baseline MADRS scores did not differ significantly between diagnostic groups, with a mean score of 27.73 in the melancholic group and 27.10 in the unspecified group (**Table 1**). In the unadjusted analysis, no statistically significant difference was observed between the two groups in terms of the reduction in MADRS scores. The changes in the mean of individual MADRS item scores over time of study were similar across both diagnostic groups (**Table 2**).

**Table 2:** Crude comparison of MADRS and individual subitems changes between baseline and at follow-up after 15 sessions of rTMS treatment.

Score change	Overall N=21^1^	Melancholic N=11^1^	Unspecified N=10^1^	p-value^2^
Overall MADRS	-13 (11)	-11 (10)	-15 (13)	0.50
Apparent Sadness	-2 (2)	-2 (1)	-3 (2)	0.30
Reported Sadness	-2 (2)	-2 (1)	-2 (2)	0.90
Inner Tension	-1 (2)	-1 (2)	-2 (2)	0.30
Inability.to.feel_change	-1 (2)	-1 (2)	-2 (2)	0.12
Pessimistic.thoughts_change	-1 (1)	-1 (1)	-1 (1)	0.70
Suicidal.thoughts_change	0 (1)	-1 (1)	0 (1)	0.20
Reduced.sleep_change	-1 (1)	-1 (2)	-1 (1)	0.80
Reduced.appetite_change	-1 (2)	-1 (1)	-1 (2)	0.80
Concentration.difficulties_change	-1 (2)	-1 (2)	-1 (2)	0.80
Lassitude_change	-2 (2)	-2 (2)	-2 (2)	0.50

1 Mean (SD).

2 Wilcoxon rank sum test.

However, after adjusting for the confounding factors, the unspecified depressive subtype was associated with a greater reduction in the overall MADRS score compared to the melancholic subtype (–9.08, 95% CI –17.69 to –0.46, p = 0.04). The unspecified depressive subtype also showed larger reductions in several specific MADRS individual items, including inner tension (–2.05, 95% CI –3.57 to –0.52, p = 0.012) and inability to feel (–1.94, 95% CI –3.10 to –0.78, p = 0.003) (**Table 3**). These item-level findings should be interpreted cautiously and considered exploratory.

**Table 3:** Results of multiple linear regression analyses showing the changes in the MADRS total score and its individual items between baseline and follow-up after 15 sessions of rTMS treatment between depressive subtypes (unspecified vs melancholic).

MADRS_change					
Predictor	Estimates	std. Beta	CI	standardized CI	p-value
Unspecified subtype	**-9.08**	**-0.81**	**-17.69 – -0.46**	**-1.58 – -0.04**	**0.040**
R2/R2 adjusted	0.604/0.434				
Apparent Sadness_change					
Unspecified subtype	-1.45	-0.81	-3.14 – 0.23	-1.76 – 0.13	0.086
R2/R2 adjusted	0.409/0.155				
Reported Sadness_change					
Unspecified subtype	-0.87	-0.5	-2.48 – 0.74	-1.41 – 0.42	0.264
R2/R2 adjusted	0.446/0.209				
Inner Tension_change					
Unspecified subtype	**-2.05**	**-1.08**	**-3.57 – -0.52**	**-1.89 – -0.28**	**0.012**
R2/R2 adjusted	0.564/0.376				
Inability to feel_change					
Unspecified subtype	**-1.94**	**-1.08**	**-3.10 – -0.78**	**-1.72 – -0.43**	**0.003**
R2/R2 adjusted	0.721/0.602				
Pessimistic Thoughts_change					
Unspecified subtype	-0.95	-0.71	-2.20 – 0.29	-1.65 – 0.22	0.123
R2/R2 adjusted	0.417/0.167				
Suicidal Thoughts_change					
Unspecified subtype	0.32	0.44	-0.36 – 1.00	-0.50 – 1.37	0.333
R2/R2 adjusted	0.503/0.290				
Reduced Sleep_change					
Unspecified subtype	0.13	0.09	-1.13 – 1.38	-0.82 – 1.01	0.833
R2/R2 adjusted	0.447/0.210				
Reduced Appetite_change					
Unspecified subtype	-0.37	-0.22	-1.71 – 0.98	-1.03 – 0.59	0.568
R2/R2 adjusted	0.565/0.379				
Concentration Difficulties_change					
Unspecified subtype	-0.57	-0.31	-1.74 – 0.59	-0.92 – 0.31	0.307
R2/R2 adjusted	0.749/0.642				
Lassitude_change					
Unspecified subtype	-0.89	-0.52	-2.36 – 0.58	-1.38 – 0.34	0.214
R2/R2 adjusted	0.554/0.363				

Models were adjusted for sex, age, administration of antidepressant, mood stabilizer medication and the baseline value of the respective dependent variable. Full models for each item are presented in **Supplementary Materials**.

Bold values indicate statistically significant results (p < 0.05).

Moreover, on average, older age (p = 0.013) was generally associated with greater overall improvement in depressive symptoms following rTMS, in contrast to the female sex that was associated with smaller improvements compared to men (**Supplementary Table 1**). Additionally, female sex appeared to be associated with less improvement for reported sadness (p = 0.047) (**Supplementary Table 3**), inability to feel (p = 0.009) (**Supplementary Table 5**) and pessimistic thoughts (p = 0.017) (**Supplementary Table 6**) compared to male sex. Older age was also associated with greater score reduction for items like inner tension (p = 0.011) (**Supplementary Table 4**), inability to feel (p = 0.012) (**Supplementary Table 5**) and lassitude (p = 0.016) (**Supplementary Table 11**). These results should be viewed as exploratory, rather than definitive effects.

## Discussion

This study aims to investigate how global depression severity and individual depressive symptoms, as measured by MADRS items, changed after 15 sessions of rTMS treatment in patients with melancholic depressive symptoms and those with unspecified depressive symptoms.

Our findings demonstrated a trend toward reduced overall depression severity following rTMS, with a reduction of 13 points of the overall MADRS score. These results align with previous literature supporting rTMS efficacy in treatment-resistant depression (10, 24–27).

Current neurobiological models suggest that high-frequency left DLPFC rTMS may engage mechanisms of particular relevance to melancholic depression by modulating the altered functional connectivity between the DLPFC and default mode network regions that characterize this subtype (14–16). Within this framework, we examined whether treatment response differed according to depressive subtype. However, in contrast to the circuit-based rationale, regression analyses showed that, under this specific 15-session rTMS protocol, improvement in total MADRS scores was greater in patients with the unspecified depressive subtype than in those with melancholic depression. These results are possibly explained by the fact that depressive symptoms with melancholic features tend to delay the response to rTMS. A recent study by Shen et al. (2025) demonstrated that symptoms related to depressed mood and anhedonia were significantly more severe in patients with melancholic depression compared to those with non-melancholic depression and required approximately two weeks to respond to rTMS treatment, longer than the response observed in patients with non-melancholic depression (28). However, since our study was exploratory, our findings need to be compared with larger sample size studies.

The MADRS items “inner tension” and “inability to feel” improved significantly more in patients in the unspecified group. Interestingly, Moyano et al. reported that melancholic patients showed greater improvement on both items following ECT (1). These findings suggest that depression severity may not be fully captured by total score changes alone, as individual symptom domains may respond differently to neuromodulatory treatments. The divergence between ECT and rTMS may reflect their distinct mechanisms of action: ECT produces widespread cortical and subcortical effects with broad neurobiological modulation (29), whereas high-frequency rTMS targeting the left DLPFC, as applied in our protocol, is thought to preferentially modulate prefrontal-limbic circuits involved in emotional and cognitive processing (30). Consequently, symptoms such as “inner tension” and “inability to feel” may reflect different underlying pathophysiological mechanisms across depressive subtypes. While these symptoms may be embedded within the broader biological dysregulation characteristic of melancholic depression and therefore respond particularly well to ECT, they may be more closely related to alterations in emotional processing and affective regulation in the unspecified depression, rendering them more responsive to rTMS. However, these interpretations remain speculative and warrant confirmation in larger studies.

These exploratory findings are primarily intended to generate hypotheses for future studies, with the aim of tailoring these interventions to each patient’s individual clinical profile. Individual characteristics, such as age, may potentially be associated with variations in symptom response, including global improvements in depressive symptoms and specific domains such as inner tension, inability to feel and lassitude. Additionally, depression subtype could play a role: not only patients with unspecified depressive symptoms showed greater overall improvements, but also exhibited greater improvement in specific items such as “inner tension” and “inability to feel”, indicating rTMS may be associated with greater improvement in this group. In contrast, melancholic symptoms appear to be less responsive to rTMS.

While neuromodulation techniques such as rTMS and ECT exhibit differentiated therapeutic effects on specific depressive symptoms, these findings should not be interpreted in isolation. Improvements in individual symptoms suggest a potentially focal and symptom-specific neuromodulatory action. However, these effects must be understood within a broader framework of response modifiers, of which depression subtype is paramount. Symptoms that appear responsive to rTMS may carry distinct neurobiological implications depending on the subtype of depression they arise in, as seen with “inner tension” and “inability to feel” in our study. Therefore, interpreting single-item effects requires a comprehensive perspective that accounts for the complexity of depressive syndrome and its clinical heterogeneity.

### Limitations

This study has several limitations that should be considered when interpreting the findings.

First, although the MADRS was administered pre- and post-treatment, in some cases the post-treatment scores had to be reconstructed from clinical records due to incomplete documentation. Combined with the absence of blinded or independent clinicians, this increases the risk of information and observer bias. This limitation is particularly relevant for item-level analyses, where measurement error may have a greater impact on individual item scores than on the total MADRS score.

Second, the small sample size substantially limits the statistical power of the subgroup and item-level analyses, particularly given the number of models and comparisons performed across the total MADRS score and individual items, increasing both type II error and false-positive findings. As a result, these exploratory analyses should be interpreted with caution and larger and more adequately powered studies are warranted to confirm potential differential responses to rTMS across depressive symptom profiles.

Third, the rTMS protocol was limited to 15 sessions, consistent with earlier trials, in which similarly short courses were effective (23). Nevertheless, shorter protocols may lead to premature classifications of late responders, particularly those with more severe depression, as non-responders, potentially underestimating treatment efficacy.

Fourth, the definition of the study groups represents an important limitation. While melancholic features were established according to DSM-5 criteria, the comparison group was defined by the absence of both melancholic and atypical features and may therefore comprise a clinically heterogeneous population. Although this approach was intended to reduce variability associated with other specifiers, the resulting asymmetry between a well-defined subtype and a residual group limits the interpretation of the findings as strictly subtype-specific. Also, three participants with low MADRS scores (12, 13 and 18) were included based on the chronicity of symptoms and their substantial functional impairment. This deviation may have introduced additional heterogeneity and affected the interpretability of treatment effects. A further limitation is that episode duration and the number and adequacy of previous antidepressant trials were not systematically recorded and could thus not be included as covariates, despite their known influence on rTMS outcomes; residual confounding by these factors cannot be excluded. Moreover, heterogeneity in the rTMS protocol was also present, as two participants received lower stimulation intensities (55% and 60% of maximal stimulator output) due to tolerability issues. This deviation from the standard protocol may have affected internal validity by introducing variability in stimulation parameters across participants.

Finally, the magnitude of the association observed in the unspecified depressive symptoms group should be interpreted cautiously. Given the limited sample size, effect size estimates may be inflated and the generalizability of these findings remains uncertain. Replication in larger, independent samples will be necessary to confirm the robustness of this association.

### Strengths

Despite the limitations outlined above, this study presents several strengths. To our knowledge, it is the first work to specifically examine the role of depressive subtypes and symptom dimensions in changes in MADRS scores following rTMS treatment, adopting an item-level approach. In line with previous findings in ECT research (1), the present study explores differential symptom-specific responses to neuromodulatory treatment.

The naturalistic design represents an additional strength, as it reflects real-world clinical practice, including patients with psychiatric and somatic comorbidities, as well as concomitant pharmacological treatments.

The use of Brainsight neuronavigation ensures precise and reproducible cortical targeting across sessions, enhancing the consistency of stimulation delivery.

Finally, the detailed clinical assessment based on item-level MADRS analysis allows for a fine-grained characterization of individual treatment responses beyond global scale scores.

## Conclusion

In conclusion, this exploratory study suggests that patients with unspecified depressive features may experience greater rTMS-related symptom improvement compared to those presenting melancholic features. These findings underscore the potential relevance of depressive subtypes in predicting treatment response, but should be interpreted with caution given the sample size and study design. More broadly, this work aligns with ongoing efforts in interventional psychiatry to tailor treatment strategies across depressive subtypes. While our findings remain preliminary and exploratory, they point to a promising line of inquiry that warrants systematic investigation in larger, controlled cohorts. Thus, this study should be regarded as an initial step in exploring the potential of item-level analyses to refine our understanding of neuromodulation effects in depression.

## Data Availability

The raw data supporting the conclusions of this article will be made available by the authors, without undue reservation.
