# Supplementary material for: Global and item-level MADRS differences between melancholic and unspecified depressive symptoms under rTMS: an exploratory analysis
Source: Front Psychiatry. 2026 Jul 24;17:1794892. doi: 10.3389/fpsyt.2026.1794892 (PMC13447313; doi:10.3389/fpsyt.2026.1794892)
Supplement: Supplementary file 1 [file DataSheet1.pdf]

Supplementary Table 1: Multiple linear regression for change in MADRS.

	MADRS_change				
Predictors	*Estimates*	*Std. Beta*	*CI*	*Standardized CI*	*p-value*
Intercept	12.54	0.39	-8.74 – 33.82	-0.12 – 0.89	0.227
Age	-0.43	-0.61	-0.75 – -0.11	-1.07 – -0.15	**0.013**
Female	10.67	0.48	2.09 – 19.25	0.09 – 0.87	**0.018**
Unspecified	-9.08	-0.81	-17.69 – -0.46	-1.58 – -0.04	**0.040**
Antidepressant	1.49	0.05	-9.41 – 12.39	-0.34 – 0.44	0.774
Mood stabilizer	-7.40	-0.29	-17.26 – 2.46	-0.67 – 0.10	0.130
Baseline MADRS	-0.29	-0.24	-0.78 – 0.20	-0.64 – 0.16	0.219
Observations	21				
R^2 / R^2 adjusted	0.604 / 0.434				

Supplementary Table 2: Multiple linear regression for change in Apparent Sadness.

	Apparent Sadness_change				
Predictors	*Estimates*	*Std. Beta*	*CI*	*Standardized CI*	*p-value*
Intercept	1.78	0.39	-2.27 – 5.82	-0.23 – 1.01	0.363
Age	-0.05	-0.40	-0.11 – 0.02	-0.95 – 0.14	0.134
Female	0.86	0.24	-0.92 – 2.64	-0.26 – 0.75	0.317
Unspecified	-1.45	-0.81	-3.14 – 0.23	-1.76 – 0.13	0.086
Antidepressant	0.01	0.00	-2.13 – 2.15	-0.48 – 0.48	0.993
Mood stabilizer	-0.67	-0.16	-2.59 – 1.24	-0.63 – 0.30	0.463
Baseline MADRS	-0.39	-0.32	-1.02 – 0.24	-0.85 – 0.20	0.210
Observations	21				
R^2 / R^2 adjusted	0.409 / 0.155				

Supplementary Table 3: Multiple linear regression for change in Reported Sadness.

	Reported Sadness_change				
Predictors	*Estimates*	*Std. Beta*	*CI*	*Standardized CI*	*p-value*
Intercept	0.86	0.24	-3.01 – 4.72	-0.37 – 0.84	0.642
Age	-0.05	-0.47	-0.11 – 0.00	-0.99 – 0.04	0.070
Female	1.72	0.50	0.03 – 3.41	0.01 – 0.98	**0.047**
Unspecified	-0.87	-0.50	-2.48 – 0.74	-1.41 – 0.42	0.264

Antidepressant	0.88	0.20	-1.19 – 2.95	-0.27 – 0.68	0.378
Mood stabilizer	-1.29	-0.32	-3.12 – 0.54	-0.77 – 0.13	0.153
Baseline MADRS	-0.43	-0.36	-1.03 – 0.17	-0.87 – 0.15	0.148
Observations	21				
R^2 / R^2 adjusted	0.446 / 0.209				

Supplementary Table 4: Multiple linear regression for change in Inner Tension.

	Inner Tension_change				
Predictors	*Estimates*	*Std. Beta*	*CI*	*Standardized CI*	*p-value*
Intercept	2.36	0.52	-1.52 – 6.24	-0.02 – 1.05	0.213
Age	-0.08	-0.63	-0.13 – -0.02	-1.10 – -0.17	**0.011**
Female	1.33	0.36	-0.23 – 2.89	-0.06 – 0.78	0.090
Unspecified	-2.05	-1.08	-3.57 – -0.52	-1.89 – -0.28	**0.012**
Antidepressant	1.13	0.24	-0.81 – 3.07	-0.17 – 0.66	0.233
Mood stabilizer	-0.07	-0.02	-1.80 – 1.67	-0.42 – 0.39	0.935
Baseline MADRS	-0.23	-0.23	-0.66 – 0.19	-0.64 – 0.19	0.260
Observations	21				
R^2 / R^2 adjusted	0.564 / 0.376				

Supplementary Table 5: Multiple linear regression for change in Inability to Feel.

	Inability to Feel_change				
Predictors	*Estimates*	*Std. Beta*	*CI*	*Standardized CI*	*p-value*
Intercept	2.57	0.51	-0.23 – 5.38	0.09 – 0.94	0.069
Age	-0.06	-0.52	-0.10 – -0.02	-0.91 – -0.13	**0.012**
Female	1.65	0.47	0.47 – 2.83	0.13 – 0.80	**0.009**
Unspecified	-1.94	-1.08	-3.10 – -0.78	-1.72 – -0.43	**0.003**
Antidepressant	0.17	0.04	-1.30 – 1.65	-0.29 – 0.37	0.806
Mood stabilizer	-1.26	-0.31	-2.58 – 0.06	-0.63 – 0.01	0.060
Baseline MADRS	-0.51	-0.42	-0.93 – -0.08	-0.76 – -0.07	**0.022**
Observations	21				
R^2 / R^2 adjusted	0.721 / 0.602				

Supplementary Table 6: Multiple linear regression for change in Pessimistic Thoughts.

	Pessimistic Thoughts_change				
Predictors	*Estimates*	*Std. Beta*	*CI*	*Standardized CI*	*p-value*
Intercept	-0.27	0.34	-3.28 – 2.74	-0.28 – 0.96	0.850
Age	-0.03	-0.36	-0.08 – 0.02	-0.92 – 0.20	0.188
Female	1.60	0.61	0.33 – 2.87	0.12 – 1.09	**0.017**
Unspecified	-0.95	-0.71	-2.20 – 0.29	-1.65 – 0.22	0.123
Antidepressant	0.34	0.10	-1.25 – 1.93	-0.37 – 0.58	0.651
Mood stabilizer	-0.29	-0.10	-1.75 – 1.16	-0.57 – 0.38	0.671
Baseline MADRS	-0.26	-0.29	-0.72 – 0.20	-0.81 – 0.22	0.246
Observations	21				
R^2 / R^2 adjusted	0.417 / 0.167				

Supplementary Table 7: Multiple linear regression for change in Suicidal Thoughts.

	Suicidal Thoughts_change				
Predictors	*Estimates*	*Std. Beta*	*CI*	*Standardized CI*	*p-value*
Intercept	0.08	-0.21	-1.61 – 1.76	-0.80 – 0.39	0.925
Age	-0.00	-0.01	-0.03 – 0.02	-0.56 – 0.54	0.980
Female	-0.02	-0.02	-0.66 – 0.61	-0.46 – 0.42	0.935
Unspecified	0.32	0.44	-0.36 – 1.00	-0.50 – 1.37	0.333
Antidepressant	-0.10	-0.06	-1.00 – 0.80	-0.55 – 0.44	0.814
Mood stabilizer	-0.38	-0.23	-1.08 – 0.33	-0.65 – 0.20	0.271
Baseline MADRS	-0.35	-0.58	-0.65 – -0.05	-1.08 – -0.08	**0.025**
Observations	21				
R^2 / R^2 adjusted	0.503 / 0.290				

Supplementary Table 8: Multiple linear regression for change in Reduced Sleep.

	Reduced Sleep_change				
Predictors	*Estimates*	*Std. Beta*	*CI*	*Standardized CI*	*p-value*
Intercept	1.24	-0.04	-2.09 – 4.58	-0.65 – 0.56	0.438
Age	-0.03	-0.37	-0.08 – 0.01	-0.87 – 0.14	0.144
Female	0.44	0.16	-1.00 – 1.88	-0.37 – 0.69	0.520
Unspecified	0.13	0.09	-1.13 – 1.38	-0.82 – 1.01	0.833
Antidepressant	0.25	0.07	-1.43 – 1.93	-0.42 – 0.57	0.755

Mood stabilizer	-1.32	-0.42	-2.75 – 0.11	-0.87 – 0.04	0.068
Baseline MADRS	-0.28	-0.37	-0.70 – 0.13	-0.91 – 0.17	0.168
Observations	21				
R^2 / R^2 adjusted	0.447 / 0.210				

Supplementary Table 9: Multiple linear regression for change in Reduced Appetite.

	**Reduced Appetite_change**				
Predictors	*Estimates*	*Std. Beta*	*CI*	*Standardized CI*	*p-value*
Intercept	0.53	0.11	-2.70 – 3.76	-0.43 – 0.64	0.728
Age	-0.03	-0.24	-0.08 – 0.02	-0.73 – 0.24	0.299
Female	1.19	0.36	-0.22 – 2.60	-0.07 – 0.80	0.092
Unspecified	-0.37	-0.22	-1.71 – 0.98	-1.03 – 0.59	0.568
Antidepressant	-0.50	-0.12	-2.19 – 1.20	-0.53 – 0.29	0.542
Mood stabilizer	-0.94	-0.25	-2.68 – 0.79	-0.70 – 0.21	0.263
Baseline MADRS	-0.40	-0.44	-0.82 – 0.02	-0.89 – 0.02	0.060
Observations	21				
R^2 / R^2 adjusted	0.565 / 0.379				

Supplementary Table 10: Multiple linear regression for change in Concentration Difficulties.

	**Concentration Difficulties_change**				
Predictors	*Estimates*	*Std. Beta*	*CI*	*Standardized CI*	*p-value*
Intercept	0.33	0.15	-2.44 – 3.10	-0.26 – 0.55	0.802
Age	0.01	0.06	-0.04 – 0.05	-0.33 – 0.46	0.739
Female	1.01	0.27	-0.14 – 2.16	-0.04 – 0.58	0.081
Unspecified	-0.57	-0.31	-1.74 – 0.59	-0.92 – 0.31	0.307
Antidepressant	-1.47	-0.31	-3.04 – 0.10	-0.65 – 0.02	0.065
Mood stabilizer	-0.41	-0.10	-1.77 – 0.94	-0.41 – 0.22	0.521
Baseline MADRS	-0.85	-0.86	-1.21 – -0.50	-1.23 – -0.50	**<0.001**
Observations	21				
R^2 / R^2 adjusted	0.749 / 0.642				

Supplementary Table 11: Multiple linear regression for change in Lassitude.

	**Lassitude_change**

Predictors	*Estimates*	*Std. Beta*	*CI*	*Standardized CI*	*p-value*
Intercept	2.41	0.25	-1.05 – 5.87	-0.31 – 0.80	0.157
Age	-0.06	-0.59	-0.11 – -0.01	-1.04 – -0.13	**0.016**
Female	1.24	0.37	-0.16 – 2.64	-0.05 – 0.78	0.077
Unspecified	-0.89	-0.52	-2.36 – 0.58	-1.38 – 0.34	0.214
Antidepressant	0.43	0.10	-1.34 – 2.20	-0.32 – 0.52	0.612
Mood stabilizer	-0.68	-0.17	-2.25 – 0.89	-0.57 – 0.23	0.370
Baseline MADRS	-0.52	-0.45	-1.00 – -0.03	-0.86 – -0.03	**0.037**
Observations	21				
R^2 / R^2 adjusted	0.554 / 0.363				

Supplementary Table 12: Descriptive of MADRS before (T0) and after (T1) the rTMS protocol.

**Characteristic**	**Overall (n = 21)**[1]	**Melancholic Subtype (n = 11)**[1]	**Unspecified Subtype (n = 10)**[1]	**p-value**[2]
MADRS T0	27 (9)	28 (6)	27 (12)	0.67
Apparent Sadness T0	4 (1)	4 (1)	4 (2)	0.67
Reported Sadness T0	4 (1)	4 (1)	4 (2)	0.39
Inner Tension T0	3 (2)	4 (2)	3 (2)	0.94
Inability to Feel T0	3 (1)	3 (1)	3 (2)	0.80
Pessimistic Thoughts T0	3 (2)	3 (1)	3 (2)	0.51
Suicidal Thoughts T0	1 (1)	1 (1)	1 (1)	0.15
Reduced Sleep T0	2 (2)	2 (2)	2 (2)	0.46
Reduced Appetite T0	2 (2)	2 (2)	2 (2)	0.79
Concentration Difficulties T0	2 (2)	2 (2)	2 (2)	0.62
Lassitude T0	3 (1)	3 (1)	4 (2)	0.18
MADRS T1	15 (11)	17 (12)	12 (9)	0.42
Apparent Sadness T1	2 (2)	2 (2)	1 (2)	0.20
Reported Sadness T1	2 (2)	2 (2)	2 (2)	0.89
Inner Tension T1	2 (2)	3 (2)	1 (2)	0.070
Inability to Feel T1	1 (2)	2 (2)	1 (1)	0.080
Pessimistic Thoughts T1	2 (2)	2 (2)	2 (2)	0.29
Suicidal Thoughts T1	1 (1)	1 (1)	1 (1)	0.25
Reduced Sleep T1	1 (2)	1 (2)	1 (2)	0.94
Reduced Appetite T1	1 (1)	1 (2)	1 (1)	0.94
Concentration Difficulties T1	1 (1)	1 (2)	1 (1)	0.57
Lassitude T1	2 (2)	1 (1)	2 (2)	0.82

[1]Mean (SD)

[2]Wilcoxon rank sum test
